# Advancements in Antioxidant-Based Therapeutics for Spinal Cord Injury: A Critical Review of Strategies and Combination Approaches

**DOI:** 10.3390/antiox14010017

**Published:** 2024-12-26

**Authors:** Yang-Jin Shen, Yin-Cheng Huang, Yi-Chuan Cheng

**Affiliations:** 1 Graduate Institute of Biomedical Sciences, College of Medicine, Chang Gung University, Taoyuan 33302, Taiwan; 2 Neuroscience Research Center, Chang Gung Memorial Hospital, Linkou, Taoyuan 333423, Taiwan; 3 Department of Neurosurgery, Chang Gung Memorial Hospital at Linkou Medical Center, Taoyuan 333423, Taiwan; 4 College of Medicine, Chang Gung University, Taoyuan 33302, Taiwan

**Keywords:** spinal cord injury (SCI), oxidative stress, antioxidant therapy, endogenous antioxidants, natural compounds, pharmacological compounds, RNA-based therapies, stem cell therapies, biomaterials, delivery systems

## Abstract

Spinal cord injury (SCI) initiates a cascade of secondary damage driven by oxidative stress, characterized by the excessive production of reactive oxygen species and other reactive molecules, which exacerbate cellular and tissue damage through the activation of deleterious signaling pathways. This review provides a comprehensive and critical evaluation of recent advancements in antioxidant-based therapeutic strategies for SCI, including natural compounds, RNA-based therapies, stem cell interventions, and biomaterial applications. It emphasizes the limitations of single-regimen approaches, particularly their limited efficacy and suboptimal delivery to injured spinal cord tissue, while highlighting the synergistic potential of combination therapies that integrate multiple modalities to address the multifaceted pathophysiology of SCI. By analyzing emerging trends and current limitations, this review identifies key challenges and proposes future directions, including the refinement of antioxidant delivery systems, the development of multi-targeted approaches, and strategies to overcome the structural complexities of the spinal cord. This work underscores the pressing need for innovative and integrative therapeutic approaches to advance the clinical translation of antioxidant-based interventions and improve outcomes for SCI patients.

## 1. Introduction

Spinal cord injury (SCI) presents a significant challenge in medical science, often resulting in irreversible neurological deficits, including paralysis, sensory loss, autonomic dysfunction, and chronic neuralgia [1,2]. The complex pathophysiology of SCI involves both primary mechanical trauma and secondary injury mechanisms, such as inflammation, apoptosis, and oxidative stress, which collectively contribute to poor clinical outcomes. Among these, oxidative stress caused by the excessive production of reactive oxygen species (ROS) and free radicals plays a pivotal role in exacerbating tissue damage while simultaneously hindering recovery [3,4]. Under normal physiological conditions, ROS, including free radicals such as superoxide anions (O_2_^•−^) and hydroxyl radicals (^•^OH), as well as non-radical species like hydrogen peroxide (H_2_O_2_), function as critical signaling molecules necessary for maintaining cellular homeostasis. However, in SCI, the overproduction of ROS triggers extensive cellular damage, promoting lipid peroxidation, protein denaturation, and DNA double-strand breaks. These events lead to cell death, tissue degeneration, and worsened functional outcomes [5,6]. To counteract the damaging effects of ROS, both endogenous and exogenous antioxidants have been explored as therapeutic agents to alleviate oxidative stress in SCI. Antioxidants neutralize ROS, limit secondary injury, and support neuronal survival and regeneration. Nevertheless, single-agent antioxidant therapies have shown limited efficacy, as they fail to address the multifaceted pathophysiology of SCI. This limitation has prompted a shift toward combination strategies, which leverage the synergistic effects of multiple antioxidants. Despite promising preclinical evidence, no antioxidant therapies have been approved for clinical use in SCI. The main barriers to clinical translation include poor bioavailability and pharmacokinetics of antioxidants, limited efficacy of single agents, and insufficient delivery and targeting strategies.

This review aims to bridge the gap between promising preclinical findings and potential clinical applications by providing a comprehensive analysis of antioxidant-based strategies for SCI. It examines the mechanisms of antioxidant action, evaluates the efficacy of various experimental approaches, highlights challenges in delivery systems, and discusses recent advancements in combination strategies. By addressing the limitations and potential solutions, this review offers a framework for integrating antioxidant-based approaches into future therapeutic strategies for SCI.

## 2. Mechanisms of ROS-Induced Damage and Endogenous Antioxidant Responses in SCI

**Figure 1:**
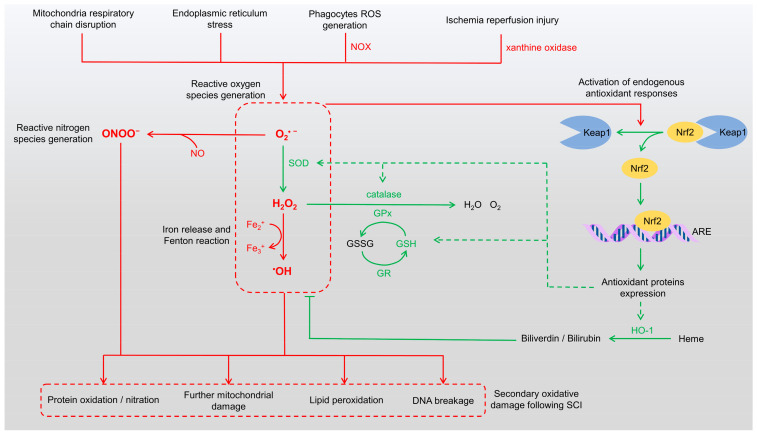
Mechanisms of ROS generation and endogenous antioxidant responses. The schematic illustrates pathways contributing to oxidative stress and their pathological consequences in SCI (red pathways). ROS are generated from sources such as mitochondrial dysfunction, endoplasmic reticulum stress, and NADPH oxidase activity in phagocytes, leading to the production of superoxide anions (O_2_^•−^), hydroxyl radicals (^•^OH), and hydrogen peroxide (H_2_O_2_). Concurrently, RNS such as peroxynitrite (ONOO^−^) are formed through interactions between ROS and nitric oxide (NO). The Keap1/Nrf2/ARE pathway (green pathways) mediates endogenous antioxidant responses, upregulating antioxidant enzymes to neutralize ROS and RNS and attenuate oxidative damage. However, excessive oxidative stress results in protein oxidation/nitration, lipid peroxidation, DNA damage, and exacerbated mitochondrial dysfunction, contributing to secondary injury and hindering recovery in SCI. ARE: antioxidant response elements, GPx: glutathione peroxidase, GR: glutathione reductase, GSH: glutathione, GSSG: glutathione disulfide, HO-1: heme oxygenase-1, NOX: NADPH oxidase, RNS: reactive nitrogen species, ROS: reactive oxygen species, SOD: superoxide dismutase.

SCI initiates a cascade of pathological events that extend beyond the initial mechanical trauma, with the overproduction of ROS playing a central role in secondary injury. These highly reactive molecules significantly exacerbate neural damage following SCI. The trauma disrupts cellular membranes, induces mitochondrial dysfunction, and activates enzymatic pathways, leading to the rapid generation of ROS [3,7]. In addition, peroxynitrite (ONOO^−^), a reactive nitrogen species (RNS), is formed by the reaction of nitric oxide with superoxide anions, further amplifying oxidative damage [8,9]. Both ROS and RNS contribute to the pathophysiology of SCI by inducing mitochondrial dysfunction, lipid peroxidation, protein oxidation/nitration, and DNA damage (Figure 1).

Mitochondria serve as a major source of ROS following SCI. The loss of cellular homeostasis impairs the electron transport chain, leading to electron leakage and the subsequent formation of superoxide anions [6,10,11]. Phagocytic cells such as neutrophils and macrophages also contribute to ROS production by generating extracellular superoxide via membrane-associated NADPH oxidase (NOX) [12,13]. Furthermore, ischemia/reperfusion injury, which occurs upon the restoration of blood flow to ischemic spinal cord tissue, exacerbates oxidative stress through the activation of ROS-producing enzymes like xanthine oxidase [14,15]. These processes not only damage cellular components but also amplify secondary injury by activating inflammatory pathways and inducing apoptosis in neurons and glial cells.

The excessive accumulation of ROS can overwhelm endogenous antioxidant defenses, leading to oxidative stress and widespread cellular damage. ROS directly attack macromolecules, including lipids, proteins, and nucleic acids, and indirectly disrupt cellular metabolic pathways and functions. Lipid peroxidation, a major mechanism of oxidative damage, involves the ROS-mediated oxidation of polyunsaturated fatty acids in membrane phospholipids. The spinal cord, enriched in polyunsaturated fatty acids, is particularly susceptible to this form of damage, which plays a critical role in the propagation of secondary injury after SCI [16,17]. ROS also inflict severe damage on DNA, particularly through hydroxyl radicals that interact with the DNA backbone, causing single- and double-strand breaks. Oxidation of nucleotide bases, especially guanine, forms 8-oxo-deoxyguanosine, a mutagenic lesion that interferes with replication fidelity [18,19]. Oxidative DNA damage activates repair mechanisms such as base excision repair and homologous recombination. However, when the damage exceeds the capacity of these repair systems, apoptosis or necrosis pathways are triggered, further exacerbating neuronal and glial cell loss and impeding spinal cord recovery.

In response to excessive ROS production, the body activates its endogenous antioxidant defense systems (Figure 1). Among these, nuclear factor erythroid 2-related factor 2 (Nrf2) serves as a central regulator of the cellular defense against oxidative stress [20,21,22]. Under basal conditions, Nrf2 is sequestered in the cytoplasm by Kelch-like ECH-associated protein 1 (Keap1), which facilitates its ubiquitination and proteasomal degradation. Upon exposure to elevated ROS levels, modifications to Keap1 result in the release of Nrf2, allowing it to translocate into the nucleus. Once in the nucleus, Nrf2 binds to antioxidant response elements (AREs), driving the transcription of numerous antioxidant and cytoprotective genes. These include key enzymes such as superoxide dismutase (SOD), catalase, heme oxygenase-1 (HO-1), glutathione peroxidase (GPx), and NADPH quinone oxidoreductase 1 (NQO1), which collectively counteract oxidative stress [23,24]. Following SCI, Nrf2 upregulation enhances antioxidant gene expression, reducing ROS levels, reducing lipid peroxidation, and protecting neurons and glial cells from apoptosis [25,26]. The Nrf2 pathway also promotes the synthesis of glutathione (GSH), bolstering cellular defenses and facilitating tissue repair and functional recovery in SCI [27]. Thus, Nrf2 is a critical modulator of oxidative stress and plays an essential role in mitigating secondary damage associated with SCI.

Enzymes activated by Nrf2 following SCI work collectively to neutralize ROS and maintain redox balance. SOD converts superoxide anions into hydrogen peroxide, which is subsequently decomposed by catalase and GPx into water and oxygen, thereby reducing the oxidative burden on injured spinal tissue [28,29]. Glutathione reductase (GR) regenerates reduced GSH from its oxidized form, glutathione disulfide (GSSG), maintaining high GSH levels necessary for GPx activity [30]. HO-1 degrades heme into biliverdin, which is further reduced to bilirubin, releasing free iron in the process. Both bilirubin and biliverdin possess antioxidant properties that neutralize ROS, while ferritin sequesters free iron to prevent oxidative damage [31]. NQO1 reduces quinone compounds, preventing the formation of semiquinone radicals, which can contribute to ROS generation [32]. Non-enzymatic antioxidants, including GSH, uric acid, melatonin, and vitamin E, also play a vital role by directly scavenging ROS, preventing excessive oxidative damage [33,34]. These endogenous antioxidants synergistically detoxify ROS and repair oxidative damage, aiding neuroprotection after SCI (Figure 1). However, excessive ROS production in SCI often overwhelms these defenses, highlighting the need for exogenous therapeutic approaches. This imbalance perpetuates oxidative stress, creating a self-sustaining cycle of inflammation, apoptosis, and further ROS production. Compounding this issue is the recruitment of immune cells such as neutrophils and macrophages, which generate additional ROS as part of the inflammatory response. The sustained oxidative stress exacerbates lipid peroxidation, protein oxidation, and DNA damage, further contributing to neuronal loss and worsening functional deficits.

## 3. Exogenous Antioxidants: Mechanisms and Roles in SCI Recovery

Antioxidant therapies for SCI are designed to alleviate oxidative stress and prevent secondary damage to the surrounding spinal cord tissue. A range of exogenous antioxidant strategies has been explored, including natural and pharmacological compounds, RNA-based therapies, stem cell therapies, and biomaterials, each exhibiting unique mechanisms of action and therapeutic potential. The following sections provide a detailed discussion of the antioxidative mechanisms, delivery methods, and therapeutic outcomes of these exogenous antioxidants in SCI models.

### 3.1. Natural Compounds as Antioxidant Therapeutics in SCI

Studies on pharmacological and natural compounds with antioxidant activities have been extensively reviewed in several articles, and we recommend that readers refer to these reviews for detailed information [35,36,37]. Here, we specifically highlight and discuss compounds that have been frequently tested in the context of SCI.

#### 3.1.1. Curcumin

Curcumin, a natural polyphenolic compound derived from turmeric, has garnered significant attention for its therapeutic potential, particularly as an antioxidant, anti-inflammatory, and anti-tumor agent [38]. Curcumin exhibits robust antioxidant properties by directly scavenging free radicals [39]. In rat SCI models, intraperitoneal injection of curcumin has been shown to enhance the activity of antioxidant enzymes such as SOD, GPx, and catalase, leading to reduced lipid peroxidation and improved functional outcomes [40,41]. Additionally, curcumin activates the Nrf2 signaling pathway and its target genes, thereby amplifying the endogenous antioxidant response to diminish oxidative damage [42,43]. Curcumin also inhibits the nuclear factor-kappa B (NF-κB) pathway, which plays a critical role in inflammatory responses. By suppressing this pathway, curcumin reduces the production of inflammatory cytokines and limits the recruitment of immune cells that exacerbate ROS production [44,45]. A rat SCI model investigating the effects of curcumin demonstrated significant improvements in neurological function and reductions in oxidative damage, with therapeutic effects showing a dose-dependent relationship [46]. Curcumin’s potent antioxidant and anti-inflammatory properties, with only minor adverse events reported in human trials across various diseases [47,48], make it a promising therapeutic agent for SCI. However, its poor bioavailability limits clinical application. Advanced delivery systems, such as nanoparticles, have been developed to improve its stability and efficacy.

#### 3.1.2. Tetramethylpyrazine (TMP)

TMP, a bioactive compound extracted from the traditional Chinese herb Ligusticum chuanxiong, has demonstrated a wide range of physiological effects, including antioxidant, anti-inflammatory, mitochondrial stabilization, and anti-apoptotic properties [49]. TMP is particularly noted for its neuroprotective effects in various neural injury models, such as cerebral ischemia, where it prevents ischemic brain damage and improves functional outcomes following middle cerebral artery occlusion in rats [50,51]. TMP also provides protection against spinal cord contusion and ischemia/reperfusion injuries [52,53,54]. The antioxidant effects of TMP include direct scavenging of free radicals [55], upregulating GSH levels via the Nrf2 signaling pathway and inhibiting HIF-1α/NOX2-mediated ROS generation. These mechanisms collectively alleviate oxidative stress and cellular apoptosis during neuronal hypoxia [56]. TMP also enhances the activities of key antioxidant enzymes, including catalase, SOD, and GPx [57,58], and reduces lipid peroxidation in spinal cord ischemia/reperfusion injury, thereby attenuating apoptotic signaling and improving neurological outcomes [58]. An animal SCI model study on TMP has demonstrated consistent reductions in oxidative stress markers and increases in antioxidant enzyme activity, contributing to improved neurological and motor recovery [59]. Clinical trials in cerebral infarction have shown that TMP generally has a high safety profile, with only a few mild adverse reactions reported, such as abdominal discomfort and dizziness [60]. However, further trials are necessary to evaluate the efficacy and safety of TMP specifically in spinal cord injury treatment. Additionally, the therapeutic efficacy of TMP as a standalone treatment is limited due to its low bioavailability and short half-life [61]. As a result, TMP is often combined with other antioxidants or incorporated into advanced delivery systems to achieve synergistic effects and enhance its potential for clinical applications.

#### 3.1.3. Vitamins

Vitamins, particularly those with antioxidant properties such as vitamin C and vitamin E, play a crucial role in inhibiting oxidative stress under normal physiological conditions and have been widely explored for therapeutic use in various diseases [62]. Vitamin C, a well-characterized water-soluble antioxidant, reduces oxidative stress through direct ROS scavenging, protecting proteins, lipids, and DNA from oxidative damage [63,64]. Beyond its antioxidative properties, vitamin C has demonstrated neuroprotective and immunomodulatory effects in neurodegenerative diseases, further supporting its therapeutic potential [65,66,67]. Vitamin E, a lipid-soluble vitamin, primarily exerts its antioxidant ability by indirect interaction with ROS [68] and neutralizing lipid peroxyl radicals, thereby interrupting lipid peroxidation and maintaining the integrity of cell membrane lipid bilayers [69,70,71]. Additionally, vitamin E has biological activities beyond its antioxidative functions, including anti-inflammatory and anti-tumorigenic properties mediated through pathways such as NF-κB, MAPK, and mTOR [72]. It also demonstrates neuroprotective effects in various in vitro models [73]. These well-documented antioxidative and neuroprotective mechanisms have prompted extensive investigation into the therapeutic potential of vitamins C and E in SCI [74]. High-dose administration of vitamins C and E has shown beneficial outcomes in rat models of SCI, leading to improved motor recovery and reduced histopathological changes in the spinal cord [75,76]. In contrast, low-dose applications of these vitamins have primarily reduced inflammatory responses without significant improvements in neurological function [76]. Combined intraperitoneal administration of vitamins C and E following SCI has demonstrated synergistic effects, significantly decreasing oxidative stress by increasing SOD activity, reducing apoptosis, and enhancing autophagy, ultimately resulting in notable functional recovery [77]. Despite these promising findings, challenges remain in translating the therapeutic potential of vitamins to clinical practice. Excessive intake of vitamin C can lead to gastrointestinal discomfort [78] and an increased risk of kidney stone formation [79], while high doses of vitamin E may disrupt vitamin K-dependent clotting [80], raising the risks of hemorrhage and all-cause mortality [81]. Addressing these concerns, alongside determining optimal dosages, refining delivery mechanisms, and reducing variability in preclinical outcomes, will be essential for advancing the clinical application of vitamin-based therapies in SCI.

#### 3.1.4. Quercetin

Quercetin, a natural flavonoid, has garnered significant attention recently for its therapeutic potential in SCI due to its potent antioxidant, anti-inflammatory, and anti-apoptotic properties [82]. Quercetin directly scavenges ROS and RNS [83,84] and enhances the expression and activity of endogenous antioxidant enzymes, including SOD1, SOD2, catalase, and GPx [85,86,87]. Furthermore, it activates the Nrf2 signaling pathway through the p38/MAPK pathway and promotes GSH synthesis [88], strengthening cellular defenses against oxidative damage. In addition to its antioxidant effects, quercetin provides mitochondrial protection by maintaining mitochondrial membrane potential, thereby reducing ROS production and lipid peroxidation [89,90]. It also inhibits microglia-mediated neuronal apoptosis by downregulating inflammatory cytokines such as TNF-α and interleukins [91] and protects other cell types, such as liver cells, from inflammatory damage through suppression of the PI3K/Akt/NF-κB signaling pathway [92]. In the context of SCI, quercetin’s therapeutic efficacy has been extensively studied in vivo, primarily using rat SCI models with intraperitoneal administration as the main delivery method [82]. In rat models, post-SCI quercetin treatment neutralizes excessive ROS and oxidative stress, reduces inflammatory cytokine levels [93], promotes the expression of antioxidant enzymes [94], and inhibits lipid peroxidation in spinal cord tissue [95]. These effects collectively facilitate improved behavioral and histopathological recovery in SCI models. Overall, quercetin’s multifunctional properties make it a highly promising therapeutic candidate for SCI. Adverse effects of quercetin are rarely reported in human applications; however, in vivo studies have demonstrated nephrotoxicity in rats subjected to long-term, high-dose administration of quercetin [96]. Ongoing research is focused on exploring its use in combination therapies and advanced drug delivery systems to enhance its efficacy and clinical applicability.

### 3.2. Pharmacological Compounds as Antioxidant Therapeutics in SCI

#### 3.2.1. Methylprednisolone and Methylprednisolone Sodium Succinate

Methylprednisolone, a widely utilized corticosteroid with potent anti-inflammatory and antioxidant properties, has been employed to treat various diseases, including SCI. It exerts its neuroprotective effects by suppressing ROS production and inflammatory cascades [97,98,99,100]. Methylprednisolone stabilizes cellular membranes by integrating into the phospholipid bilayer, thereby preventing lipid peroxidation [98,101]. Additionally, it enhances the activity of endogenous antioxidant enzymes such as SOD and GPx [102,103], while inhibiting pro-inflammatory cytokines, including TNF-α and interleukins, which are closely linked to oxidative stress and ROS generation [104,105,106]. A derivative of methylprednisolone, methylprednisolone sodium succinate, is chemically modified with a sodium succinate ester group to improve its pharmacological properties. This derivative has been extensively evaluated in various preclinical SCI models, demonstrating significant therapeutic potential in acute SCI [101,107]. Preclinical successes supported the use of methylprednisolone in clinical settings, further validated by the National Acute Spinal Cord Injury Study (NASCIS) trials [108,109]. Methylprednisolone was, at one point, the only treatment widely applied in clinical practice for acute SCI. However, subsequent clinical trials and studies yielded mixed results. While moderate neurological improvements were observed, these benefits were often accompanied by significant side effects, including increased risks of infection and gastrointestinal bleeding [110,111]. Consequently, the routine use of high-dose methylprednisolone for SCI patients is no longer recommended in contemporary clinical practice. These outcomes underscore both the therapeutic potential and the limitations of corticosteroids in SCI treatment, highlighting the need for more targeted and safer pharmacological approaches.

#### 3.2.2. Minocycline

Minocycline, a clinically available tetracycline antibiotic, has demonstrated significant antioxidant, anti-inflammatory, and anti-apoptotic properties in addition to its well-known antimicrobial effects. These properties have been extensively studied in various neurodegenerative and neural injury models, including SCI [112,113,114]. Minocycline’s ability to target multiple secondary injury mechanisms—such as excessive oxidative stress, inflammation, glutamate excitotoxicity, cellular calcium influx, and mitochondrial dysfunction—positions it as a promising therapeutic agent for alleviating secondary injury processes following SCI [115,116]. Minocycline treatment in SCI models has been shown to enhance levels of GSH and increase the activity of antioxidant enzymes such as SOD and GPx, while significantly reducing lipid peroxidation [116,117]. Additionally, minocycline exerts neuroprotective effects by suppressing microglial activation, reducing oxidative stress, and reducing neuronal apoptosis [118,119,120]. It also stabilizes mitochondrial membranes, thereby decreasing ROS release from mitochondria and preserving mitochondrial function [121,122]. Furthermore, minocycline’s metal-chelating properties provide protection against iron-mediated neurotoxicity and membrane lipid peroxidation [123,124,125]. This capability is particularly relevant in SCI, where excess iron contributes to oxidative stress and neurodegeneration. Minocycline also inhibits NOX, a critical enzyme involved in ROS production by activated microglia and neutrophils [126,127]. While a phase II clinical trial found no significant difference in functional outcomes between placebo and minocycline-treated patients, the drug was deemed safe, with minimal adverse events reported even at high doses [128]. The multifaceted antioxidant and neuroprotective properties of minocycline make it a compelling candidate for addressing secondary injury after SCI. Its integration with other antioxidants or delivery systems is being increasingly explored to enhance its efficacy and therapeutic potential in preclinical and translational studies.

#### 3.2.3. Metformin

Metformin, a widely used first-line treatment for diabetes mellitus, has demonstrated antioxidant properties that expand its therapeutic potential beyond glucose regulation. Metformin mitigates oxidative damage in various disease models by enhancing the activity of antioxidant enzymes such as SOD and GPx, reducing inflammatory cytokine levels [129,130], and increasing concentrations of antioxidant molecules like thiols and vitamin C [131]. Additionally, it protects against radiation-induced DNA damage through mechanisms such as ion chelation and oxidative DNA repair [132]. By activating the AMPK/Nrf2 signaling pathway, metformin amplifies endogenous antioxidant responses, providing neuroprotection in conditions such as cerebral ischemia and Alzheimer’s disease [133,134]. Metformin has been extensively studied in SCI animal models, particularly in rats, with intraperitoneal injection as the primary delivery route. Post-SCI metformin treatment has been shown to enhance functional recovery and neuronal survival [135]. It also promotes axonal regeneration by stabilizing microtubules through the PI3K/Akt signaling pathway, which preserves structural integrity and supports axonal elongation. Furthermore, metformin stabilizes mitochondrial membranes and bolsters antioxidant defenses by activating Nrf2 signaling and its downstream targets, including HO-1 and NQO1, reducing oxidative damage in injured spinal cord tissues [136]. In addition to these effects, metformin has been found to significantly reduce neuronal ferroptosis via HO-1 signaling in SCI models, further highlighting the importance of HO-1 in its antioxidant actions [137]. These properties—ranging from oxidative stress regulation to cellular repair promotion—position metformin as a promising therapeutic candidate for SCI. Approved by the Food and Drug Administration for managing type II diabetes mellitus, metformin is generally well-tolerated, with mild-to-moderate digestive disturbances being the most common side effects, and rarer occurrences of hypoglycemia, anemia, or lactic acidosis [138]. However, challenges remain, including its limited targeting efficiency within the spinal cord and the difficulty of translating in vivo results into clinical applications.

#### 3.2.4. N-Acetylcysteine (NAC)

NAC, an acetylated derivative of L-cysteine, is commonly used as a mucolytic agent for respiratory diseases and as a detoxifying agent in acetaminophen overdose [139]. Its effectiveness in acetaminophen poisoning stems from its ability to replenish hepatic GSH levels [140]. As a precursor to GSH, NAC has attracted considerable interest for its potential to treat diseases associated with elevated oxidative stress, including various neurological disorders [141,142,143]. In rodent SCI models, NAC has been extensively studied, typically administered through intraperitoneal injection. NAC treatment significantly reduces lipid peroxidation and increases SOD activity, demonstrating its capacity to prevent oxidative damage after SCI. Its antioxidant effects have been shown to be comparable to those of methylprednisolone in a rat SCI model [144]. Additionally, NAC preserves mitochondrial function and mitigates associated oxidative stress, reducing neuronal loss, demyelination, and inflammation. These effects contribute to improved locomotor recovery following SCI [145,146]. Despite these promising findings, isolated use of NAC has shown limited therapeutic benefits in some studies. For instance, a recent investigation reported only slight improvements in locomotor activity, lesion size, and spinal cord morphology, with no significant effect on interlimb coordination [147]. In contrast, combining NAC with early decompression surgery in a rat SCI model significantly improved histological and functional outcomes by reducing inflammation and apoptosis [148]. This suggests that NAC’s therapeutic efficacy may be enhanced when used in combination with other interventions. Together, these findings highlight NAC’s potential as an antioxidant therapy for SCI.

### 3.3. RNA-Based Therapies for Antioxidant Treatment

#### 3.3.1. MicroRNAs (miRNAs)

miRNAs are endogenous small non-coding RNAs that regulate gene expression by base-pairing with specific RNA sequences, resulting in the repression or degradation of their mRNA targets [149]. miRNAs play a crucial role in modulating the oxidative stress response across various disease models [150,151,152,153]. In SCI, several miRNAs have been identified as key regulators of the antioxidant response by targeting genes encoding antioxidant enzymes, highlighting their essential role in maintaining ROS homeostasis [154]. For instance, transcriptomic analysis has demonstrated that miR-137 exerts a protective role following SCI. Transfection of miR-137 into primary cultured spinal cord cells from mice reduced oxidative stress and inflammation, potentially via the degradation of NEUROD4 [155]. Following SCI, hyperactivation of Nrf2 in astrocytes has been shown to exert neuroprotective effects by upregulating miR-145-5p, which enhances the expression of antioxidant enzymes such as SOD2 and NQO1 [156]. In a rat SCI model, antisense miR-486 was directly injected into spinal cord tissue to knock down miR-486, resulting in significant motor function improvement. The decreased miR-486 levels induced *NEUROD6* expression, which upregulated key antioxidative proteins, including thioredoxin-like 1 and GPX3, promoting ROS scavenging and neuroprotection [157]. Additionally, miR-99a agomir, a chemically modified RNA molecule mimicking endogenous miR-99a, was infused directly into the SCI lesion site via microinjection, improving allodynia and motor function recovery while reducing ROS levels in injured tissues [158]. Intrathecal administration of miR-7a mimics alleviated mitochondrial dysfunction, increased SOD-1 and HO-1 expression, reduced oxidative stress, and enhanced motor function recovery after SCI [159]. Infusion of miR-23b into injured mouse spinal cords targeted neuropathic pain by downregulating NOX4 expression, protecting motor neurons and astrocytes from ROS-mediated apoptosis, and improving neuropathic pain indices [160]. Mulberrin, a natural compound derived from the root of Ramulus Mori, diminishes oxidative stress and inflammation following rat SCI by downregulating miR-337 and modulating Nrf2 signaling [161]. These studies highlight miRNA as a promising therapeutic approach for modulating oxidative stress and promoting neuroprotection in SCI.

#### 3.3.2. Small Interfering RNAs (siRNAs)

siRNAs can be either exogenous double-stranded RNAs introduced into cells to selectively silence specific genes or endogenous small RNAs processed from double-stranded RNA precursors within cells. Both forms function through the RNA interference (RNAi) pathway, binding to target mRNAs with perfect or near-perfect complementarity to induce mRNA cleavage and degradation [162,163]. Artificially synthesized or delivered siRNAs are particularly valuable for targeting genes implicated in oxidative stress and have been evaluated in various disease models [164,165,166]. In SCI, *NOX4* siRNA injected into mouse spinal cords significantly increased thioredoxin-like 1 expression, reduced inflammatory markers, and improved neuropathic pain after SCI [160]. Knocking out *TREM1*, an innate immune receptor associated with inflammation, enhanced motor function, decreased oxidative stress markers, and increased antioxidant protein expression in injured spinal cord tissue. In vitro transfection of *TREM1* siRNA into microglial and astrocytic cells further demonstrated that TREM1 silencing reduced oxidative stress via HO-1 signaling [167]. However, the application of siRNA as an antioxidant therapy in SCI remains limited, highlighting the need for further research to fully explore its therapeutic potential and efficacy.

#### 3.3.3. Long Noncoding RNAs (lncRNAs)

LncRNAs, RNA molecules over 200 nucleotides long, regulate gene expression at transcriptional, translational, and epigenetic levels by interacting with nucleic acids and proteins [168]. They recruit chromatin-modifying complexes, form DNA hybrid structures to influence chromatin accessibility [169], act as miRNA sponges to regulate mRNA expression [170], and modulate mRNA splicing and protein turnover through lncRNA–protein complexes [171]. These numerous functions lead to their emergence as important therapeutic targets in various physiological and pathological contexts. In SCI, significant changes in lncRNA expression have been implicated in various processes, including the regulation of oxidative stress via miRNA modulation [172]. For instance, the lncRNA MALAT1 exhibits antioxidative and neuroprotective roles in multiple neurological disease models [173,174,175]. Delivered through viral vectors, MALAT1 exerts neuroprotective effects in SCI by sequestering miR-204 in a rat model of spinal cord ischemia/reperfusion injury [176] and inhibiting miR-125b-5p-induced microglial M1 polarization [173]. MALAT1 also promotes Nrf2 nuclear translocation and attenuates spinal cord tissue damage in SCI rats treated with low-dose lipopolysaccharide [177], highlighting its critical role in oxidative stress regulation and cell survival. Other lncRNAs also show altered expression following SCI. For example, lncRNA CASC9 is downregulated, while lncRNA H19 is upregulated in rat SCI models [178,179]. Overexpression of CASC9 in LPS-induced PC12 cells reduced apoptosis, lipid peroxidation, inflammatory cytokines, and lactic acid while increasing Nrf2 and HO-1 protein expression, highlighting its protective role against oxidative stress and inflammation [178]. In contrast, inhibition of lncRNA H19 with siRNA in lipopolysaccharide-stimulated spinal neurons reversed pathological changes, reducing ROS production and apoptosis while improving cell viability. Notably, the therapeutic effect of lncRNA H19 inhibition was counteracted by miR-370-3p upregulation, suggesting that the lncRNA H19/miR-370-3p pathway governs oxidative stress and neuronal apoptosis following SCI [179].

Despite their therapeutic potential, significant challenges remain in the development of RNA-based antioxidants for SCI. These include rapid degradation of single-stranded RNA molecules and off-target effects causing unexpected side effects. Effective delivery systems that prevent RNA degradation and enhance cellular selectivity are essential for unlocking the therapeutic potential of RNA therapies in SCI.

### 3.4. Antioxidant Properties of Stem Cell Therapy

Stem cell therapies have been extensively studied for SCI treatment due to their neuroprotective properties and ability to promote regeneration in damaged neural tissues [180]. Beyond their regenerative capacity, stem cells also exhibit significant antioxidant effects. They alleviate oxidative stress through multiple mechanisms: releasing antioxidant enzymes such as SOD, catalase, and GPx to neutralize ROS and reduce lipid peroxidation and DNA damage [181,182]; secreting neuroprotective factors like brain-derived neurotrophic factor (BDNF), nerve growth factor (NGF), and vascular endothelial growth factor (VEGF) to attenuate oxidative stress and support cellular repair [183,184]; modulating microglia and astrocyte activation to shift the inflammatory response toward a neuroprotective, less oxidative profile [185,186]; and enhancing endogenous antioxidant defenses by upregulating antioxidant genes and activating intrinsic protective pathways, including Nrf2 signaling [184,187]. Collectively, these mechanisms reduce oxidative damage, preserve cellular integrity, and promote functional recovery after SCI.

#### 3.4.1. Bone Marrow Mesenchymal Stem Cells (BM-MSCs)

BM-MSCs are multipotent stem cells found in adult bone marrow that support hematopoiesis and contribute to the regeneration of bone and connective tissue [188]. BM-MSCs have gained recognition for their potential in SCI treatment due to their robust paracrine signaling and antioxidant properties [189,190]. Their therapeutic effects are primarily attributed to paracrine actions rather than direct differentiation into neurons, enhancing neuroprotection by activating antioxidant and anti-inflammatory responses and secreting neurotrophic factors such as NGF and BDNF, which promote axon growth and synaptic connections [191,192,193]. In neural injury models, transplanted BM-MSCs reduce oxidative stress by secreting antioxidant enzymes such as SOD, catalase, and GPx, which neutralize ROS and RNS and prevent lipid peroxidation [194,195,196]. Studies in a bleomycin-induced pulmonary fibrosis model demonstrated that BM-MSCs exert antioxidant effects through Nrf2 signaling activation [197]. Additionally, BM-MSCs improve functional recovery following SCI by promoting tissue repair, reducing inflammation, and restoring motor function [198,199,200,201,202]. Combination therapies further enhance their efficacy. For example, BM-MSCs combined with plumbagin, a natural naphthoquinone compound, injected into injured rat spinal cords improved motor function, reduced edema, and exhibited antioxidant effects by increasing SOD levels and Nrf2 expression [203]. In another study, BM-MSCs overexpressing miR-200a activated Nrf2 signaling, reduced oxidative stress, and enhanced antioxidant enzyme activities, including SOD and catalase, while upregulating Nrf2 target proteins HO-1 and NQO1, resulting in improved locomotor recovery rat SCI model [204].

#### 3.4.2. Adipose-Derived Mesenchymal Stem Cells (AD-MSCs)

AD-MSCs, isolated from adipose tissue through minimally invasive techniques, are another type of multipotent stem cells with high proliferation capacity and strong regenerative, immunomodulatory, and antioxidant properties [189,190]. Their accessibility and therapeutic potential make AD-MSCs a promising option for SCI treatment [205]. In SCI, AD-MSCs promote neural repair through various mechanisms, including replacing lost neurons after priming for neuronal differentiation and transplantation [206], secreting neurotrophic factors such as BDNF, VEGF, and ciliary neurotrophic factor (CTNF) [207,208], promoting angiogenesis [209], and modulating the immune response via Notch signaling [210]. To exert antioxidant effects in oxidative stress models, AD-MSCs enhance SOD activity and upregulate antioxidant enzymes such as NQO1 and GPx, reducing ROS, RNS, and lipid peroxidation levels [211,212,213]. Compared to BM-MSCs, AD-MSCs demonstrate similar antioxidant effects but with greater accessibility and a lower risk of immune rejection. In a canine SCI model, intravenously delivered AD-MSCs improved hindlimb function and neuronal survival, exhibiting superior antioxidative and anti-inflammatory effects compared to high-dose methylprednisolone, without adverse effects [214]. Additionally, AD-MSCs overexpressing HO-1, designed to boost antioxidant potential and resilience against oxidative stress, significantly improved motor function, reduced inflammation and astrogliosis, and enhanced neuroregeneration in SCI models [215,216]. Pre-conditioning AD-MSCs with melatonin, a hormone with versatile antioxidant properties, further enhanced engraftment and differentiation into neuronal lineages in a rat SCI model, resulting in better functional recovery [206]. These findings underscore the therapeutic promise of AD-MSCs in SCI treatment and highlight opportunities for further modifications to optimize their antioxidant effects.

In antioxidant research, MSCs excel over other stem cell types due to their exceptional immune modulation and robust paracrine functions. However, neuronal-lineage and pluripotent stem cells, despite less explored antioxidant properties, play a more direct role in neuronal replacement and integration into spinal cord neural circuits, which is essential for spinal cord regeneration. While their intrinsic antioxidant effects are limited, maintaining redox balance is critical for their survival and function. Reducing oxidative stress and enhancing endogenous antioxidant defenses could further improve their therapeutic efficacy in SCI. The following sections briefly discuss the antioxidant properties and therapeutic potential of stem cells other than MSCs, emphasizing how their modification can mitigate oxidative stress and support SCI recovery.

#### 3.4.3. Neural Stem/Progenitor Cells (NSPCs)

NSPCs are lineage-specific stem/progenitor cells present in the developing embryonic central nervous system and, to a limited extent, in the adult CNS. In adults, NSCs have been identified in regions such as the subventricular zone of the lateral ventricles, the subgranular zone of the hippocampus, and the central canal of the spinal cord [217,218]. These cells can differentiate into primary neural lineages, including neurons [219,220,221], astrocytes, and oligodendrocytes [222,223,224], facilitating neurogenesis and remyelination following CNS injuries. Numerous studies on NSPC transplantation in animal models of SCI have shown improved motor function, reduced lesion size, and enhanced tissue preservation [225,226,227]. In neural injury and neurodegenerative models, NSPCs have been observed to upregulate SOD2 expression in neurons and reduce ROS production in response to neurotrophic factors [183,228]. The application of NSPC-derived secretomes in a rat SCI model demonstrated antioxidant and anti-inflammatory properties, leading to improved locomotor recovery and reduced neuropathic pain [229], highlighting their potential as antioxidant agents for SCI. Moreover, activation of Nrf2 signaling in NSPCs enhances the expression of antioxidant enzymes, protecting these cells from oxidative stress and promoting their survival while preserving self-renewal capabilities [230]. These findings emphasize the promise of NSPCs as a therapeutic option for SCI, particularly due to their dual role in regeneration and antioxidative defense.

#### 3.4.4. Embryonic Stem Cells (ESCs)

ESCs are pluripotent stem cells derived from the inner cell mass of the blastocyst. They exhibit self-renewal capacity and can differentiate into all cell types of the three germ layers, making them a powerful tool in regenerative medicine [231]. In the context of SCI, undifferentiated ESCs are less commonly used due to their tumorigenic potential [232]. Instead, ESC-derived neural progenitors and oligodendrocyte progenitors are more frequently employed for SCI treatment [233]. In rodent SCI models, transplanted ESC-derived neural progenitors differentiate into neuronal and glial lineages, promoting axonal growth, remyelination, and angiogenesis [234,235,236]. ESC-derived oligodendrocyte progenitors have demonstrated substantial remyelination effects [237,238]. The role of intrinsic redox regulation and mitochondrial function in governing the characteristics of ESCs has been highlighted in several studies [239,240]. Expression of antioxidant enzymes such as SOD2 and catalase, which modulate redox status, is critical for determining whether ESCs maintain self-renewal or undergo neuronal differentiation [241].

#### 3.4.5. Induced Pluripotent Stem Cells (iPSCs)

iPSCs function as pluripotent stem cells derived from reprogrammed adult somatic cells, such as skin or blood cells. This approach reduces the risk of immune rejection and avoids ethical concerns associated with ESCs [242,243]. In rodent SCI models, transplantation of human iPSC-derived NSPCs has led to their differentiation into neuronal and glial cells, resulting in the reconstruction of neuronal circuits with existing neurons, enhanced angiogenesis, axonal regeneration, and notable motor improvement [244,245]. Transplantation of iPSC-derived oligodendrocyte progenitors during the early stages of SCI has also been shown to promote remyelination of damaged axons and facilitate functional recovery [237,246]. Redox balance is critical for the survival and differentiation of iPSCs. The abundant expression of endogenous antioxidant enzymes in iPSCs reduces intracellular oxidative stress and maintains genomic integrity essential for stemness [247,248]. A combination therapy comprising human iPSC-derived NSPCs and a polyacetal-curcumin nanoconjugate has been developed, wherein curcumin protects iPSC-derived NSPC from H_2_O_2_-induced oxidative damage. This combination was injected directly into the lesion site following rat SCI, inhibiting glial scar formation while promoting the survival of motoneurons and axons [249].

#### 3.4.6. Extracellular Vesicles (EVs)

EVs have gained considerable attention due to their promising therapeutic potential. These small, lipid bilayer-enclosed vesicles are secreted by nearly all cell types and are present in various bodily fluids, including plasma, urine, and cerebrospinal fluid [250]. Stem cell-derived EVs, in particular, possess significant therapeutic potential [251,252]. EVs facilitate cell-cell communication by transporting bioactive molecules such as nucleic acids, proteins, and lipids [253]. Among EV subtypes, exosomes (50–200 nm in diameter) have garnered interest due to their low immunogenicity, high biocompatibility, and ability to preserve the biological activity of their cargo [254,255]. Additionally, exosomes exhibit strong chemotactic properties, enabling them to localize to injury sites and cross the blood-spinal cord barrier (BSCB), facilitating tissue repair [256,257]. In a rat SCI model, intravenously delivered NSPC-derived EVs reduced neuronal apoptosis, microglial activation, and neuroinflammation by enhancing autophagy and modulating apoptotic gene expression [258]. Systemically delivered MSC-derived exosomes facilitated SCI repair and functional recovery by downregulating inflammatory cytokines [259] and regulating apoptotic signaling via the miR-21-5p/FasL axis [260]. Beyond their anti-apoptotic and immunomodulatory effects, EVs are closely associated with oxidative stress [261]. EVs not only produce antioxidants and detoxify ROS but are also regulated by oxidative stress, which affects their production and cargo composition [262]. Pro-inflammatory or pro-oxidant conditions have been shown to induce EV release in vitro [263]. For example, exosomes derived from MSCs under hypoxic conditions reduced ROS accumulation, DNA damage, and apoptosis in a HIF-1α-dependent manner in a rat colitis model [264]. EVs also deliver molecules such as antioxidant enzymes, miRNAs, and proteins that scavenge ROS directly or activate antioxidative cascades in target cells [261]. The Nrf2/HO-1 axis has been identified as a key mediator of the antioxidative and anti-inflammatory effects of MSC-derived EVs [265,266]. In the context of SCI, AD-MSC-derived exosomes injected intravenously in a rat model promoted neuronal repair and angiogenesis by activating the Nrf2/GPX4 pathway and inhibiting endothelial ferroptosis [267]. Intrathecal delivery of MSC-derived exosomes significantly inhibited ferrous iron production, lipid peroxidation, ROS generation, and ferroptosis, improving neurological outcomes [268]. Hydrogel-loaded MSC-derived exosomes implanted directly into rat SCI lesions reduced inflammation and oxidative stress, resulting in improved motor function and preserved urinary function [269]. NSPC-derived exosomes were also shown to inhibit neuronal apoptosis post-SCI by activating autophagy via miR-374-5p and its target genes [270]. Despite these promising findings, the molecular mechanisms and signaling pathways underlying the therapeutic effects of EVs in SCI remain largely unexplored. Standardizing isolation protocols and characterizing active molecules within EVs are critical steps to ensure reproducibility and efficacy before translating EV-based therapies to clinical applications in SCI patients [271].

### 3.5. Biomaterials in Antioxidant-Based Therapeutics

Recent studies have increasingly focused on the application of biomaterials in SCI therapy, highlighting their potential as direct ROS scavengers or catalysts and their remarkable capacity for loading and controlled release of antioxidants. Leveraging their intrinsic enzyme-like antioxidant activities, carbon nanomaterials and metal-based nanoenzymes have been applied in various disease models, including SCI, to counteract excessive oxidative stress in injured tissues. Additionally, biocompatible polymers provide steady, continuous, and prolonged antioxidant release while serving as pro-survival platforms for hosting stem cells post-transplantation into injured spinal tissue. Nanoparticles and liposomes further enhance targeted antioxidant delivery through systemic circulation. The following sections discuss various biomaterials, focusing on their antioxidant mechanisms, therapeutic efficacy, delivery strategies, and associated challenges.

#### 3.5.1. Biomaterials as Antioxidant Agents

Carbon nanomaterials have gained significant attention for their antioxidative potential, owing to unique physicochemical properties such as electrical conductivity, mechanical strength, and enzymatic activity [272]. Among these, fullerenes, spherical carbon molecules with a hollow structure, have demonstrated exceptional free radical scavenging abilities [273]. In a mouse model of chronic autoimmune encephalomyelitis, a fullerene derivative linked to an NMDA receptor antagonist effectively targeted oxidative stress and excitotoxic effects. This approach significantly reduced axonal loss, demyelination, oxidative injury, and immune cell infiltration into spinal cord tissues [274]. Carbon dots are nanoscale particles with unique photochemical properties and excellent biocompatibility that demonstrate strong ROS-scavenging capabilities [275]. Selenium-doped carbon dots have been shown to protect neuronal and astrocytic cell lines from H_2_O_2_-induced oxidative damage [276]. In a rat SCI model, these particles reduced inflammation, glial scar formation, and oxidative stress, enhancing locomotor recovery [277]. Carbon nanotubes, cylindrical graphene-based structures, are noted for their strength, conductivity, and ability to promote neural growth and circuit formation [278]. Their electron acceptability also makes them potent radical scavengers [279,280], demonstrating neuroprotection through antioxidant and immunomodulation properties [281,282]. In a rat SCI model, injecting multi-walled carbon nanotubes loaded with ivermectin reduced oxidative stress and inflammation, improving locomotor activity and neuropathic pain [283]. However, concerns about bioaccumulation, chronic toxicity, and immune activation remain challenges for their clinical translation, emphasizing the need for further research into safer, biodegradable nanomaterials.

Metal-based nanozymes are nanoparticles with intrinsic enzyme-like catalytic activities that mimic the functions of natural oxidoreductases or hydrolases and have shown substantial potential in scavenging ROS and reducing oxidative stress [284,285,286]. Cerium oxide (CeO_2_) nanoparticles are particularly well studied for their mimetic properties resembling SOD, catalase, and peroxidase. These nanoparticles alternate between Ce^3+^ and Ce^4+^ oxidation states to neutralize ROS and prevent oxidative damage [287]. In a rat model of contusion SCI, local injection of CeO_2_ nanoparticles into the lesion cavity inhibited the expression of genes involved in ROS generation, apoptosis, and inflammation while reducing lesion size and promoting motor function recovery [288]. Manganese (Mn) is a critical cofactor for several metalloenzymes, including SOD2 (Mn-SOD), glutamine synthetase, and arginase [289]. Mn-based nanoenzymes have been widely developed, exhibiting enzymatic activities that mimic those of GPx and SOD [290,291]. For instance, chitosan-modified manganese dioxide nanoparticles encapsulating resveratrol—a natural polyphenolic compound with antioxidant and anti-inflammatory properties—have been administered intravenously in SCI mice. This approach provided sustained antioxidant protection by reducing ROS levels and lipid peroxidation while enhancing antioxidant defenses through increased SOD and GPx activity in vivo [292]. Mn-porphyrins have also been extensively employed to alleviate oxidative stress following SCI. Intrathecal delivery of Mn-porphyrins significantly reduced oxidative damage while promoting the survival of neurons and glial cells [293]. Notably, this treatment demonstrated superior therapeutic outcomes compared to intravenous methylprednisolone administration [294,295]. Iron oxide (Fe_2_O_3_) nanoparticles, known for their peroxidase-like activity, catalyze the breakdown of H_2_O_2_, thereby reducing oxidative stress [296]. In a rat SCI model, Fe_2_O_3_ nanoparticles embedded in an agarose gel were implanted into the transection lesion and paired with an external electromagnetic field. This approach led to significant functional recovery and a reduction in lesion volume. In vitro experiments demonstrated effective cellular uptake of the nanoparticles and a marked decrease in H_2_O_2_-mediated oxidative stress [297]. Zinc oxide (ZnO), another metal-based nanozyme with peroxidase-like properties, has also shown promise in SCI treatment [298]. ZnO nanoparticles exhibited neuroprotective effects in an in vitro axotomy model by reducing oxidative stress and apoptosis through the activation of the PI3K-Akt signaling pathway [299]. When locally administered, ZnO nanoparticle-loaded hydrogel effectively reduced ROS production and the inflammatory response in the injured spinal cord of mice, providing neuroprotection and facilitating functional recovery [300]. Gold nanomaterials, with enzyme-mimicking activities resembling those of SOD, peroxidase, and catalase, have also demonstrated potential in SCI treatment [301]. In a mouse SCI model, intravenous injection of gold nanoclusters stabilized by zinc modification exhibited significant antioxidant properties. These nanoclusters reduced ROS-induced neuronal apoptosis and inflammation, improved ventral motor neuron survival, and caused minimal systemic toxicity [302]. Beyond these metallic nanoparticles, a hollow-structured single-atom cobalt nanozyme was developed with broad-spectrum capabilities to neutralize multiple ROS and RNS molecules. Its porous structure enabled the encapsulation and controlled release of minocycline, enhancing neuroprotection and motor function recovery in SCI rats [303].

Despite the well-recognized antioxidant properties of carbon nanomaterials and metal-based nanozymes, their application in SCI remains underexplored, with only a limited number of studies investigating their therapeutic potential. Challenges to their clinical translation persist, particularly due to the dual roles of metal-based nanozymes as both ROS scavengers and ROS producers [304,305,306]. Environmental factors such as pH variability [307], local ROS concentrations [308], and other conditions can influence the behavior of these nanozymes, sometimes resulting in unpredictable effects. In addition, carbon nanomaterials, while capable of scavenging ROS to alleviate oxidative stress, may, under specific stimuli, promote ROS generation [275,309,310,311], potentially causing unintended oxidative damage to non-target tissues. Furthermore, the lack of complete biodegradability of both metal-based nanozymes and carbon nanomaterials poses additional challenges. Their accumulation in organs during metabolism raises concerns about chronic toxicity and heightened immune responses. To address these limitations, further research is needed to optimize the design, functional modifications, and delivery methods for these nanomaterials, ensuring their efficacy and biocompatibility in clinical applications.

#### 3.5.2. Biomaterials and Delivery Strategies for Antioxidant Therapy

Nanoparticles, ranging in size from 1 to 100 nanometers, possess unique features such as a high surface area, tunable properties, and small size, making them highly effective in the treatment of various diseases. Their applications include targeted tissue delivery, enhanced drug transport, and modulation of injury environments. Antioxidants such as curcumin and resveratrol face challenges of poor bioavailability due to limited absorption, rapid metabolism, and low systemic availability when administered orally or systemically [312]. Nanoparticles provide a promising solution by encapsulating these compounds, enabling enhanced penetration across the BSCB [313], shielding them from rapid metabolism, and improving their systemic bioavailability [314,315]. The following sections examine the antioxidant properties of various nanoparticles, along with their therapeutic cargo, delivery routes, and targeting mechanisms.

Intravenous administration remains the most common method for delivering nanoparticles in SCI therapy. For instance, double-coated nanozymes, where SOD1 was incorporated into a poly-ethylene glycol (PEG)–polylysine polycation complex and then coated with PEG–polyglutamic acid polyanion polymers, have been shown to prolong the circulation of active SOD1 in the bloodstream. These nanozymes reduced inflammation, oxidative stress, and edema, ultimately improving locomotor function recovery in a rat SCI model [316]. TMP-loaded nanoparticles conjugated with the HIV transactivator of transcription (TAT) peptide and human serum albumin facilitated neutrophil uptake and BSCB penetration. This system improved TMP solubility and bioavailability, resulting in reduced oxidative stress and inflammation, as well as enhanced locomotor recovery in SCI rats [317]. Nanoparticles co-loaded with curcumin and retinoic acid in a bovine serum albumin self-assembly system were applied to a mouse SCI model. These nanoparticles scavenged ROS effectively, induced macrophage polarization toward regenerative phenotypes, reduced inflammatory responses, promoted neuronal differentiation, and minimized scar tissue formation [318]. Another approach used zein-based nanoparticles encapsulating metformin and functionalized with the CAQK peptide, which targets chondroitin sulfate proteoglycans upregulated in SCI lesions. These inflammation-targeting nanoparticles exhibited potent anti-inflammatory, antioxidant, and neuroprotective effects, leading to increased neurofilament density, improved axonal myelination, reduced glial scarring, and enhanced motor function recovery [319]. Additionally, multi-functionalized selenium nanoparticles have been developed, incorporating a polysaccharide-protein complex for enhanced stability and the PG-6 peptide, which specifically targets matrix metalloproteinases-2 and -9, abundant in the SCI microenvironment. These nanoparticles were loaded with TMP and monosialotetrahexosylganglioside 1 to counteract excessive ROS and oxidative stress. This system demonstrated strong antioxidant and neuroprotective effects, reducing mitochondrial dysfunction, preventing apoptosis, and significantly improving functional recovery in a rat SCI model [320].

Local administration is an effective method for delivering nanoparticles, involving their direct injection into the injury site. This approach ensures a high concentration of nanoparticles at the target area, providing localized and sustained release of therapeutic agents while minimizing systemic exposure and reducing the risk of degradation and toxicity. For instance, polydopamine nanoparticles loaded with rapamycin, an mTOR inhibitor, were locally injected into spinal cord lesions in a rat SCI model. These nanoparticles exhibited antioxidant effects by inhibiting mTOR-mediated ROS overproduction and, in vivo, reduced cavity size, improved hind limb coordination, and enhanced neurogenesis and tissue regeneration [321]. Another example is a microcomposite system comprising a poly-lactic-co-glycolic acid (PLGA) polymer core containing methylprednisolone, coated with a polydopamine shell. This system targeted inflammatory cytokines, provided localized drug delivery, and protected spinal cord tissue in a rat SCI model [322]. Poly-caprolactone (PCL)-based nanoparticles loaded with minocycline were administered locally during the acute phase of SCI in mice, reducing pro-inflammatory microglial responses, sustaining a pro-regenerative environment, and supporting long-term functional recovery [323]. Localized delivery systems have also been employed for siRNA therapies, addressing challenges such as poor cellular uptake and rapid enzymatic degradation. For example, mesoporous silica nanoparticles were designed to deliver IRF5-targeted siRNA with ROS-responsive degradation and controlled release. This system promoted the transformation of macrophages into a pro-regenerative phenotype [324]. Chitosan nanoparticles loaded with siRNA targeting inducible nitric oxide synthase were used to reduce harmful nitric oxide production, minimizing secondary injury and promoting spinal cord recovery by limiting RNS release [325].

Oral administration of nanoparticles has also been explored as a promising alternative, offering flexible dosing schedules, improved patient compliance, and stable systemic concentrations. Selenium nanoparticles administered orally in a rat SCI model improved immune cell profiles and reduced histological markers of inflammation at the injury site [326]. In addition, oral gavage of selenium nanoparticles derived from selenium-enriched Proteus mirabilis cultures were delivered via oral gavage in a rat SCI model, demonstrating antioxidant and anti-inflammatory effects. The treatment reduced oxidative stress and lipid peroxidation levels, enhanced nerve regeneration, and improved hind limb motor function [327].

Liposomes, larger than nanoparticles and typically ranging from 50 to 500 nm in diameter, are vesicles composed of a phospholipid bilayer. Their stability, biocompatibility, ease of modification, high drug-loading capacity, and amphipathic properties—allowing encapsulation of both hydrophilic and hydrophobic drugs—have made them one of the most widely explored nanocarriers [328]. Similar to nanoparticles, liposomes are often conjugated with TAT peptides to facilitate penetration across cells and the BSCB. In a rat SCI model, TAT-modified liposomes demonstrated significantly higher accumulation at the lesion site [329]. Additionally, TAT- and PEG-conjugated magnetic polymeric liposomes containing superparamagnetic nanoparticles were found to target injury sites through magnetic force, enhancing BSCB penetration [330]. These findings suggest that TAT peptide modification and magnetic attraction offer feasible targeting strategies for liposome-based delivery in SCI. Further innovations have employed the natural targeting ability of macrophages toward injury sites. For example, macrophage membrane-camouflaged liposomes, isolated from macrophages and administered intravenously, selectively accumulated at the SCI site in a mouse model. When loaded with minocycline, these liposomes exhibited strong therapeutic effects, including anti-inflammatory and anti-pyroptotic actions, suppressed glial scar formation, reduced axonal necrosis [331], and decreased calcium-induced ROS generation and subsequent lipid peroxidation [332]. A complex delivery system was tested in a rat SCI model, combining oxidation resistance 1 (OXR1) plasmids condensed in vitamin E succinate-grafted polylysine nanoparticles encapsulated within liposomes. The OXR1 protein, which regulates genes involved in antioxidant defense, reduced oxidative damage. This system facilitated stable OXR1 plasmid transfection with minimal cytotoxicity, alleviating oxidative stress and promoting functional recovery by upregulating Nrf2, HO-1, catalase, and SOD1 [333].

Hydrogels are hydrophilic polymers capable of retaining substantial water while maintaining structural integrity, making them highly suitable for SCI applications due to their excellent biocompatibility, high water content, and similarity to the extracellular matrix. Studies have shown that alginate hydrogels support neurite outgrowth and alleviate oxidative stress in primary cultured neurons [334]. When implanted into a rat hemimyelonectomy SCI model, alginate hydrogels promoted functional recovery with reduced fibrous scar formation at the lesion site [335]. Innovative ROS-scavenging hydrogels have been developed by crosslinking thioketal-containing hyperbranched polymers with methacrylate hyaluronic acid. These hydrogels effectively neutralized hydrogen peroxide and superoxide anions in vitro. When loaded with BM-MSCs and growth factors and implanted into a rat SCI model, they attenuated oxidative damage and inflammatory cytokines, reduced fibrotic and glial scarring, and enhanced neurogenesis [336]. Antioxidant-loaded hydrogels, such as those containing curcumin or quercetin, have also been widely studied for their ability to optimize controlled release and localized delivery of antioxidants [337,338,339,340]. Several studies have evaluated the therapeutic efficacy of antioxidant-loaded hydrogels in SCI models. For instance, in a rat model of unilateral cervical contusion SCI, which impairs respiratory function, a minocycline-loaded agarose/chitosan hydrogel administered via subdural injection reduced spinal cord damage and preserved diaphragm motor neuron innervation, thereby maintaining respiratory function [341]. In another study, a rapamycin-loaded PVA hydrogel layered over polylactic acid electrospun fibers encapsulating BDNF was applied to a rat hemisection SCI model. The dynamic borate-ester crosslinked hydrogel exhibited responsive ROS-scavenging effects, while rapamycin facilitated autophagy by enhancing autophagosome formation. This system reduced exogenous ROS levels and stimulated endogenous antioxidant responses by upregulating Nrf2, HO-1, and related antioxidant enzymes. It simultaneously inhibited glial scar formation and neuronal apoptosis, while promoting autophagy, axonal growth, and neuroprotection [342]. Other examples include chitosan hydrogels loaded with curcumin and selenium nanoparticles, which were locally implanted at the SCI site. This combination synergized antioxidant effects, reducing edema and inflammation during the acute phase and decreasing necrotic neurons and inflammatory cell infiltration. However, it also induced reactive astrocytosis during the late phase of SCI [343]. TMP-loaded electroconductive hydrogels have also shown significant antioxidant properties and neuroprotection in a rat SCI model [344]. Stem cell-derived EVs are frequently incorporated into hydrogels to enhance therapeutic outcomes. A hydrogel composed of N-acryloyl glycinamide and gelatin methacrylate containing MSC-derived EVs and tannic acid demonstrated significant restoration of motor function and urinary function after implantation into the injured spinal cord. This system reduced oxidative DNA damage, lipid peroxidation, and inflammatory cytokines, facilitating tissue repair [269]. Moreover, a polylysine-based hydrogel designed for sustained release of AD-MSC-derived EVs and aminoguanidine reduced oxidative damage and scar tissue formation, and promoted remyelination and axonal regeneration at the lesion site [345].

Injectable hydrogels have been highlighted as a promising approach for SCI therapy, offering minimally invasive delivery and precise localization to the injury site. For example, a thermosensitive hydrogel containing MnO_2_/apoferritin nanoparticles encapsulating astragaloside, an antioxidant from Astragalus membranaceus, was directly injected into the lesion in a rat contusion SCI model. This treatment elevated GSH levels, enhanced SOD activity, and alleviated oxidative stress-induced ferroptosis, leading to improved neuronal survival, reduced tissue damage, and enhanced hindlimb motor function [346]. Furthermore, NSCs encapsulated in a ROS-scavenging gelatin hydrogel composed of CeO_2_ nanoparticles promoted neural differentiation and integration of transplanted cells, effectively reducing oxidative stress and inflammation in SCI tissue [347]. In another study, a hydrogel loaded with NSC-derived exosomes facilitated neurite regrowth and improved neurological function recovery in a mouse SCI model through the exosomal delivery of miR-34a-5p [348].

In addition to hydrogels, various polymeric formulations have been explored for SCI repair, including scaffolds, nanofibers, and conduits, many of which have been specifically designed to target oxidative stress [349,350]. For instance, a hybrid nanofiber scaffold composed of PCL and polysialic acid encapsulating methylprednisolone was directly transplanted into the lesion area following rat SCI. This system effectively reduced the release of inflammatory cytokines and the expression of apoptotic proteins, while also preventing axon demyelination and reactive gliosis [351]. In a rat hemisection SCI model, insulin-like growth factor-1 (IGF-1) and BDNF were incorporated into biodegradable PLGA/graphene oxide electrospun nanofibers. This nanofiber membrane protected NSCs from H_2_O_2_-induced oxidative stress and promoted neuronal differentiation in vitro. When applied to the surface of the injured spinal cord, the membrane reduced cavity formation and increased neuron density at the injury site, resulting in improved locomotor recovery [352]. Chitosan/polyvinyl alcohol (PVA) nanofibers encapsulating genistein, a natural phytoestrogen with antioxidant and anti-inflammatory properties, were used to cover spinal cord lesions in another hemisection SCI model. This treatment enhanced SOD activity while reducing nitric oxide levels, lipid peroxidation, and inflammatory markers, ultimately mitigating oxidative stress and inflammation within the injured tissue [353]. To overcome the harsh environment with excessive ROS SCI, which threatens post-transplanted MSC survival, a composite scaffold system featured an outer ROS filter constructed from PVA crosslinking with a ROS-responsive linker tetramethylpropane-1,3-diaminium and the inner porous hydrogel housing MSC spheroids was implanted into transected rat spinal cord. The ROS filter rapidly scavenged excess ROS, shielding MSCs from oxidative damage and upregulating Nrf2 and HO-1 expression during the acute phase. The enhanced paracrine effect of MSCs promoted neuronal repair and significantly improved electrophysiological and motor function recovery in the chronic phase [354].

These polymer-based systems offer multifunctional capabilities, including structural support for axon guidance, creating a conducive environment for stem cells, intrinsic antioxidative properties, or efficient antioxidant delivery. Collectively, they represent promising candidates for integrating various therapeutic approaches to enhance SCI repair and recovery.

## 4. Challenges for Clinical Translation and Future Directions

### 4.1. Administration Routes and Drug Delivery Issues

Despite the promising efficacy of antioxidant therapies, demonstrated in preclinical studies, several challenges remain in translating these approaches into clinical practice. A significant hurdle lies in the delivery of antioxidants, as their therapeutic potential is most effective during the acute phase of injury when access to spinal cord tissue is inherently difficult. Systemic delivery, including nanoparticle-based formulations and liposomal encapsulations as discussed previously, represents an innovative approach for enhancing bioavailability and targeting. However, the efficiency of targeting and the controlled release of antioxidants through these systems remain limited due to physical and physiological barriers. To address these challenges, direct delivery of antioxidants into the spinal cord has been extensively explored. Intrathecal delivery, which administers therapeutic agents directly into the cerebrospinal fluid, bypasses the BSCB and enables more efficient access to spinal cord tissue [249,268,293]. This technique offers a significant advantage in achieving localized and effective drug concentrations. Future advancements may include the use of intrathecal catheters, which allow for the steady and repeated infusion of antioxidants to the injury site, potentially enhancing controlled and targeted interventions [355,356]. Additionally, innovative delivery methods such as convection-enhanced delivery have shown potential for SCI treatment. Convection-enhanced delivery employs pressure-driven bulk flow generated through specialized catheters, facilitating more extensive and uniform distribution of therapeutic agents within spinal cord tissue [357,358]. Another promising strategy involves injectable hydrogels, which have garnered considerable interest for their ability to encapsulate therapeutic agents. These hydrogels can be administered directly into the lesion site without requiring invasive surgery, offering a minimally invasive alternative to traditional implants [346,347].

**Figure 2:**
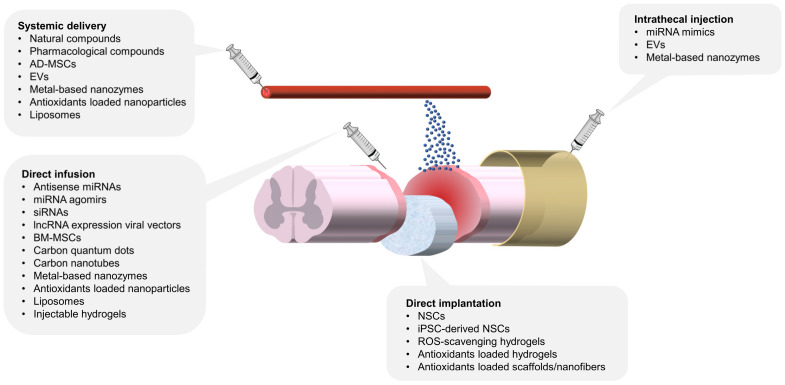
Multiple antioxidant therapies for SCI and their delivery routes. This schematic illustrates various delivery methods for antioxidant strategies targeting spinal cord injuries. The delivery methods include direct infusion, direct implantation, systemic delivery, and intrathecal injection. Direct infusion and implantation focus on localized therapeutic action, while systemic delivery emphasizes immunomodulation and targeting SCI lesions. Intrathecal injection, though less explored in experimental models, holds significant clinical potential due to well-established techniques for spinal tapping and drug administration. AD-MSC: adipose-derived mesenchymal stem cell, BM-MSC: bone marrow mesenchymal stem cell, NSC: neural stem cell, EVs: extracellular vesicles.

Figure 2 provides an overview of various antioxidant therapies under investigation, highlighting their respective delivery routes, including systemic administration, direct infusion, implantation, and intrathecal injection. Each method offers distinct advantages in addressing the challenges of targeting SCI lesions and optimizing therapeutic efficacy.

### 4.2. The Complexity of Spinal Cord Tissue

The spinal cord’s intricate anatomical structure, diverse cellular composition, and highly organized neural circuits present significant challenges for effective SCI treatment. Following injury, this complexity is further exacerbated by the formation of necrotic cavities, glial scars, and cystic spaces [359,360]. These structural irregularities hinder the uniform distribution of therapeutic agents, making it difficult for systemically administered antioxidants to penetrate and reach all damaged areas. The diversity of cell types within the spinal cord also complicates antioxidant treatments. Neurons, oligodendrocytes, astrocytes, microglia, and infiltrating immune cells exhibit distinct responses to oxidative stress and injury. Neurons and oligodendrocytes are particularly vulnerable to ROS-induced damage, which leads to neural death and demyelination [361,362,363]. Concurrently, oxidative stress stimulates M1 polarization of macrophages, enhancing phagocytic function and debris clearance. However, a controlled level of ROS can promote the polarization of microglia toward the M2 phenotype, which supports tissue repair and neuroprotection [364,365]. Therefore, antioxidant therapies must be carefully designed to achieve both optimal distribution within the spinal cord and a delicate balance of ROS levels across different cell types, creating an environment conducive to tissue regeneration and functional recovery.

To address the complexity of spinal cord injury, advanced stereoscopic biostructures have been developed. Organoids, which mimic the tissue architecture and cellular diversity of an organ, represent a significant advancement in this area [366]. Spinal cord organoids derived from iPSCs provide a physiologically relevant in vitro model to study the neural networks and cellular interactions within the spinal cord, as well as to screen experimental therapies [367,368]. These organoids can be utilized to model oxidative damage after SCI, enabling high-throughput testing of antioxidant treatments. In therapeutic applications, organoids derived from reprogrammed human astrocytes have been transplanted into mice SCI lesions, where they demonstrated neuronal differentiation and integration into host neural circuits [369]. This highlights the potential of organoids as a therapeutic strategy for SCI repair. Another promising approach is 3D bioprinting, which offers precision in creating complex structures and combines cells with biomaterials in a spatially controlled manner [370]. For example, a dual-network hydrogel was 3D printed with a temperature-responsive outer layer and an N-cadherin-modified inner layer, targeting temporal regulation of oxidative stress and neuroregeneration in SCI. The hydrogel facilitated the release of ROS scavengers to protect endogenous neural stem/progenitor cells during the acute phase of injury, while supporting their migration and neuronal differentiation during the later phase [371]. These advanced bioengineering technologies offer innovative solutions to address the challenges posed by the spinal cord’s complexity. By combining antioxidant therapies with organoid and 3D bioprinting technologies, researchers can overcome the limitations of current treatment strategies, paving the way for more effective and precise SCI interventions.

## 5. Conclusions

The spinal cord is particularly susceptible to oxidative stress and free radical-mediated damage due to its high lipid content and active oxygen metabolism. Despite extensive basic and clinical research into the pathophysiology of secondary injury after SCI, the complexity of these mechanisms continues to hinder the development of effective therapies. This review has highlighted several antioxidants with potential therapeutic roles in SCI treatment, encompassing a wide range of approaches, including antioxidative molecules, stem cells, and biomaterials. Each therapeutic approach demonstrates significant antioxidative activity but faces distinct challenges in SCI treatment, such as adverse effects and insufficient efficacy as standalone regimens, limited bioavailability and biocompatibility, poor targeting of the injured spinal cord, and inadequate penetration across the BSCB. These limitations underscore the necessity of combining multiple antioxidant strategies to achieve synergistic and complementary effects, with particular emphasis on the role of biomaterials as a platform for integrating diverse antioxidant therapies.

While numerous antioxidant therapies have demonstrated promising results in experimental SCI models, no effective antioxidant-based treatments are currently available for clinical use. The translation of these findings into clinical practice faces significant challenges, including issues related to the timing and delivery of antioxidants and the complex structural and cellular composition of the spinal cord. This review has proposed potential strategies to address these obstacles, such as innovative delivery systems and advanced bioengineering technologies. Nevertheless, further research and development are required to refine multiplex antioxidant therapies and facilitate the translation of preclinical success into clinical applications, ultimately improving outcomes for SCI patients.

## Data Availability

No new data were created in this article.

## Abbreviations

AD-MSC	adipose-derived mesenchymal stem cell.
ARE	antioxidant response elements.
BDNF	brain-derived neurotrophic factor.
BM-MSC	bone marrow mesenchymal stem cell.
BSCB	blood-spinal cord barrier.
CeO_2_	cerium oxide.
CTNF	ciliary neurotrophic factor.
ESC	embryonic stem cell.
EVs	extracellular vesicles.
Fe_2_O_3_	iron oxide.
GPx	glutathione peroxidase.
GR	glutathione reductase.
GSH	glutathione.
GSSG	glutathione disulfide.
H_2_O_2_	hydrogen peroxide.
HO-1	heme oxygenase-1.
iPSC	induced pluripotent stem cell.
Keap1	Kelch-like ECH-associated protein 1.
lncRNA	long noncoding RNA.
miRNA	microRNA.
Mn	manganese.
Mn-SOD	manganese superoxide dismutase.
NAC	N-acetylcysteine.
NF-κB	nuclear factor-kappa B.
NGF	nerve growth factor.
NOX	NADPH oxidase.
NQO1	NADPH quinone oxidoreductase 1.
Nrf2	nuclear factor erythroid 2-related factor 2.
NSC	neural stem cell.
O_2_^•−^	superoxide anions.
^•^OH	hydroxyl radicals.
ONOO^−^	peroxynitrite.
PCL	poly-caprolactone.
PEG	poly-ethylene glycol.
PLGA	poly-lactic-co-glycolic acid.
PVA	poly-vinyl alcohol.
RNS	reactive nitrogen species.
ROS	reactive oxygen species.
SCI	spinal cord injury.
siRNA	small interfering RNA.
SOD	superoxide dismutase.
TAT	transactivator of transcription.
TMP	Tetramethylpyrazine.
TNF-α	tumor necrosis factor-α.
VEGF	vascular endothelial growth factor.
ZnO	zinc oxide.
